# Genome‐enabled prediction for sparse testing in multi‐environmental wheat trials

**DOI:** 10.1002/tpg2.20151

**Published:** 2021-09-12

**Authors:** Leonardo Crespo‐Herrera, Reka Howard, Hans‐Peter Piepho, Paulino Pérez‐Rodríguez, Osval Montesinos‐Lopez, Juan Burgueño, Ravi Singh, Suchismita Mondal, Diego Jarquín, Jose Crossa

**Affiliations:** ^1^ International Maize and Wheat Improvement Center (CIMMYT) Km. 45, Carretera México‐Veracruz, El Batán, Texcoco, Edo. de México, CP El Batan 56130 Mexico; ^2^ Univ. of Nebraska–Lincoln Lincoln NE 68583 USA; ^3^ Biostatistics Unit Univ. of Hohenheim Fruwirthstrasse 23 Stuttgart 70599 Germany; ^4^ Colegio de Postgraduados Montecillos, Edo. de Mexico Mexico; ^5^ Univ. de Colima Colima Mexico

## Abstract

Sparse testing in genome‐enabled prediction in plant breeding can be emulated throughout different line allocations where some lines are observed in all environments (overlap) and others are observed in only one environment (nonoverlap). We studied three general cases of the composition of the sparse testing allocation design for genome‐enabled prediction of wheat (*Triticum aestivum* L.) breeding: (a) completely nonoverlapping wheat lines in environments, (b) completely overlapping wheat lines in all environments, and (c) a proportion of nonoverlapping/overlapping wheat lines allocated in the environments. We also studied several cases in which the size of the testing population was systematically decreased. The study used three extensive wheat data sets (W1, W2, and W3). Three different genome‐enabled prediction models (M1–M3) were used to study the effect of the sparse testing in terms of the genomic prediction accuracy. Model M1 included only main effects of environments and lines; M2 included main effects of environments, lines, and genomic effects; whereas the remaining model (M3) also incorporated the genomic × environment interaction (GE). The results show that the GE component of the genome‐based model M3 captures a larger genetic variability than the main genomic effects term from models M1 and M2. In addition, model M3 provides higher prediction accuracy than models M1 and M2 for the same allocation designs (different combinations of nonoverlapping/overlapping lines in environments and training set sizes). Overlapped sets of 30–50 lines in all the environments provided stable genomic‐enabled prediction accuracy. Reducing the size of the testing populations under all allocation designs decreases the prediction accuracy, which recovers when more lines are tested in all environments. Model M3 offers the possibility of maintaining the prediction accuracy throughout both extreme situations of all nonoverlapping lines and all overlapping lines.

## Core Ideas

- Genome‐enabled prediction for sparse field testing of lines was used.
- Allocation design of lines was tested in some environments.
- Breeding lines in multi‐environments trials with genomic prediction were analyzed.

**Abbreviations**
BLUE: best linear unbiased estimator
CIMMYT: International Maize and Wheat Improvement Center
CV: cross‐validation
E: environmental main effects
G: genomic main effects
GE: genotype × environment interaction
GP: genome‐based prediction
iid: independently, identically, and normally distributed
L: line main effects
MET: multi‐environmental trial
NOL: nonoverlapping
OL: overlapping
TPE: target population environment.

## 1 INTRODUCTION

Global food production is being challenged by the continuous deterioration of natural resources, a continuously growing population with significant dietary changes, and the drastic fluctuations occurring in climate conditions. Wheat (*Triticum aestivum* L.) is a staple food crop that provides about 20% of the energy intake in the human diet and is grown on over 215 million ha (USDA, 2020). Given its importance, it is paramount to increase wheat productivity in the face of these constraints. A major method used to increase wheat grain yield, yield stability, disease resistance, and nutritional and end‐use quality is by means of genetic improvement.

Coping with the current global scenario requires making crop breeding processes more efficient, in such a way that the targeted genetic gains are achieved. One aspect of the breeding processes is yield testing. When evaluating for yield under different environments, or when looking for stable lines or cultivars with reliable yield under such varying conditions, the phenomenon of genotype × environment interaction (GE) becomes important. This phenomenon corresponds to a genotype‐specific response when environmental conditions change. The variation in this GE response can be used to select genotypes adapted to specific target population of environments (TPEs) specifying both the climate and the production environment. To investigate the specific GE of the germplasm, extensive field‐testing and multi‐environmental trials (METs) are usually conducted. The METs help assess the stability of cultivar across environments and specific GE interactions, which can be exploited when breeding for a certain TPE.

Within the last two decades, genome‐based prediction (GP) of genetic values (Meuwissen et al., 2001) has revolutionized plant and animal breeding (Crossa et al., 2010, 2011, 2017; Hayes et al., 2009; Jannink et al., 2010). The principle of GP is to use densely distributed molecular markers across the entire genome, usually in a linear regression model, to predict the performance of individuals with known genotypes but unknown phenotypes. For research purposes, the accuracy of predictions is usually evaluated using some form of random cross‐validation (CV) that splits the data set into a training and a testing set. The objective is to predict the phenotypes of the testing set with the information (phenotypic and genomic) of the training set. The predicted and observed values in the testing set are then compared to assess the model's performance. The random CV attempts to emulate what would occur in real GP decisions under the same environmental stimuli. For example, random scheme CV1 used in this paper (described in more detail later, along with other schemes; Burgueño et al., 2012; Jarquín et al., 2014) represents the situation in which a set of newly developed breeding lines or cultivars were never tested in any of the environments of the multi‐environmental trial. This represents a case of sparse testing where a percentage of these lines were never observed in any of the environments and must be predicted. Random scheme CV2 (Burgueño et al., 2012; Jarquín et al., 2014) represents a less extreme case of sparse testing because some lines were observed in some environments but not in others.

The literature on the design of efficient multi‐environment field trials for plant breeding is extensive (Cullis et al., 2020) and should be considered in the design of experiments within the context of genome‐based assisted breeding. For example, for early generation testing, the *p*‐rep design of Cullis et al. (2006) is currently established in early testing of plant breeding, whereas Williams et al. (2011) combined the *p*‐rep designs with the augmented designs (where only checks are repeated). Piepho and Williams (2006) investigated the effect of genetic correlated family‐based lines for developing efficient design of field trials in plant breeding programs; this is important in the context of genome‐based multi‐environment trials when genomic similarity between lines assists breeders in defining appropriate training populations.

**Core Ideas**

- Genome‐enabled prediction for sparse field testing of lines was used.
- Allocation design of lines was tested in some environments.
- Breeding lines in multi‐environments trials with genomic prediction were analyzed.

Recently, Tolhurst et al. (2019) fitted single‐step multi‐environment trials to plant breeding MET datasets incorporating molecular marker data. The authors suggested that several articles on genome‐enabled predictions fail to adequately accommodate joint modeling of the nongenetic sources of variation and environmental variation in a single‐step analysis. However, Piepho et al. (2012) and Damesa et al. (2017, 2019) demonstrated that the incorporation of all relevant sources of variation can also be achieved in a weighted stage‐wise fashion in plant breeding and genome‐based predictions, yielding results as precise as single‐stage analyses with little or no differences in weighted versus unweighted analyses.

Sparse testing in plant breeding and in genome‐enabled prediction can be emulated through different random CV schemes. This implies modifying the original multi‐environment breeding trial system into a system where not all breeding lines are sown in all environments because costs and factors like seed, land, and water availability might impede observing all genotypes in all environments. Depending on the stage of the breeding pipeline, in the late stages it might be desirable to expand the set of environments to conduct field evaluations. In these cases, the variability of the environment can be captured through a reduced set of lines tested in these environments, leveraging this information to effectively predict the untested genotypes.

The question is how to establish a multi‐environmental trial system that will be economically feasible, saving land and other resources, without adversely affecting the precision with which the performance of breeding lines or cultivars is assessed, predicted, and selected. In genomic‐assisted breeding, the information provided by the molecular markers is leveraged to help breeders to predict unobserved lines in specific environments. Although in most cases it is not possible to evaluate all genotypes in all environments for the aforementioned reasons, observing some of these individuals offers the possibility of assessing the marker alleles in all environments and the marker × environment interaction as well. Hence, the information of the response patterns of the markers and the marker × environment interaction can be leveraged to improve the predictive ability of the unobserved lines in the environments. Thus, sparse testing plays an important role in making multi‐environment trials economically feasible while not affecting much of the field trial precision.

By using genome‐enabled prediction when modeling GE, the nonobserved genotype × environment combinations can be better predicted and thus the overall costs of the testing can be reduced. Recently, Jarquín et al. (2020) stated that the accuracy of predicting unobserved lines is influenced by (a) the number of overlapping (OL) lines between environments, that is, lines that are field‐tested in several environments (OL lines in environments); (b) the number of environments where each line is grown; and (c) the prediction model used. Sparse evaluation can include extreme cases of (a) nonoverlapping (NOL) lines between environments, all lines tested in different environments; and (b) lines completely OL across environments, all lines field evaluated in all the environments; and (c) varying numbers of different or NOL/OL genotypes (Jarquín et al., 2020). The results obtained by Jarquín et al. (2020) in maize (*Zea mays* L.) multi‐environment trials showed that the genome‐based model including GE captured more phenotypic variation than the models that did not include this component and provided higher prediction accuracy than other genomic prediction models that did not include GE when applying them to multiple sparse testing designs. Thus, the results obtained using sparse testing designs can lead to substantial savings in testing resources when using appropriate genome‐based models to optimize selection accuracy.

For the reasons outlined above, the main objective of this research was to study the genome‐enabled prediction accuracy of different sparse testing allocation systems and, given a total number of wheat lines and a total number of environments, determining how different numbers of breeding lines can be arranged in different environments by a system that systematically assigns a certain number of NOL and OL lines to different environments. Jarquín et al. (2020) had a similar allocation of NOL/OL lines in environments for an efficient genome‐based scheme in multi‐environments maize trials.

The ratio of number of lines to number of environments gives the number of NOL lines that can be accommodated in each environment or the maximum number of NOL lines per environment. We studied three general allocation designs (OL lines are used to connect environments): (a) all NOL (zero OL lines in environments), which implies that different subsets of genotypes were assigned to completely different environments, thus the design is disconnected with respect to the genotype × environment classification based on phenotypic information; in this way, all lines of interest were assigned to different environments and observed once in any of the environments and thus those nonobserved lines in environment combinations needed to be predicted;(b) completely OL (zero NOL) lines where a subset of all of the lines (tested/training set) of interest are tested in all environments and the rest of the lines (prediction set) were never observed in any environments and thus needed to be predicted; and (c) a combination of the two previous cases, in which a certain number of NOL/OL lines were distributed in the environments; this represents various proportions of NOL/OL lines. The study used three extensive wheat data sets (W1, W2, W3).

Given the GE context in which the data sets were generated, we fitted three different models for each of the NOL/OLO general cases to determine which prediction strategy more accurately addresses the problem of different sparse allocation of tested lines. Those models were (a) only environmental (E) and line main effects (L) (no molecular marker information or any interaction) (Model M1 = E+L); (b) E, L, and genomic main effects (G) (Model M2 = E+L+G); (c) E, L, G, and GE (Model M3 = E+L+G+GE).

## 2 MATERIALS AND METHODS

### 2.1 Wheat experimental multi‐environment data sets

For this study, we used three extensive wheat data sets from International Maize and Wheat Improvement Center (CIMMYT), which correspond to evaluations of elite line trials during the periods of 2013–2014 (W1; 942 unique lines), 2014–2015 (W2; 1,008), and 2016–2017 (W3; 1,038) observed in six environments during each period. In addition, molecular marker information was available for the lines evaluated in these periods as follows: 6,071 SNPs for W1, 5,963 SNPs for W2, and 8,312 SNPs for W3. The environments included in this study represent main mega‐environments (or TPEs) (Crespo et al., 2021) with different levels of irrigation: irrigated, drought; two different sources of heat: early heat, late heat; and two different planting systems: bed and flat. These six environments were established in the field of the main CIMMYT wheat experiment station in Cd. Obregon (Mexico).

The six different environments in which the wheat lines of W1, W2, and W3 were tested are

1. Beds 5 irrigations: Trials conducted on raised beds with full irrigation management (optimal), about 500 mm of available water; optimal planting date (late November to mid‐December).
2. Flat 5 irrigations: Trials planted on flat land with full irrigation (optimal), about 500 mm of available water; optimal planting date.
3. Beds 2 irrigations: Trials conducted on raised beds with partial irrigation, about 260 mm of available water; optimal planting date.
4. Flat drought: Trials planted on flat land with severe drought, about 180 mm of available water; optimal planting date.
5. Beds early heat: Trials planted in late October (nonoptimal planting date) subject to heat stress and fully irrigated, about 500 mm of available water.
6. Beds late heat: Trials planted in late February (nonoptimal planting date), subject to heat stress and fully irrigated, about 500 mm of available water.

### 2.2 Allocation design for sparse testing

The different allocation schemes depend on the number of NOL/OL wheat genotypes in each environment. The OL lines are used to connect environments (Jarquín et al., 2020).

### 2.3 Small example for allocation design

A small example (Table 1) is provided for illustration purposes to show how the testing set and prediction set are composed before and after the inclusion of common genotypes across environments. Here, we were interested in the performance of 24 lines in six environments; thus the total number of lines‐in‐environments is 24 × 6 = 144. If there are available resources to test only 24 of these line × environment combinations, the next question is how to distribute these among the six environments. Here, the total number of line × environment combinations for the testing set (set to observe in fields) is 24, generating 24 phenotypic values. The NOL scheme (bolded cells in Table 1) shows all 24 genotypes in only one environment each. In our case, four different genotypes are tested in each one of the six environments. Thus, the OL/NOL ratio becomes (0:4) because within each environment we have zero OL genotypes. A modified design allowing OL sets is performed by observing some of the genotypes (2) across all of the environments, thus reducing the number of unique genotypes to only 14. The cells with “x” represent the genotypes that are part of the testing set. In this case, only 14 unique genotypes are tested in fields, 12 of which are observed once and two are observed six times each. Thus, the new testing set would be composed of 24 phenotypic values that correspond to 12 unique genotypes and another set of 12 phenotypes that correspond to only two genotypes observed six times each (24 = 12 × 1+ 6 × 2) resulting in a OL/NOL ratio of (2:2) because within each environment two of the observed genotypes were also observed across all environments while the remaining two were exclusively observed in these two environments but not in the others.

**TABLE 1:** Toy example. Sparse allocation design for 24 genotypes (1‐24) in six environments (1‐6) where two different genotypes are nonoverlapped in six environments (6 × 2 = 12) and two genotypes are repeated/observed across six environments (2 × 6 = 12). The nonoverlap/overlap ratio is 2:2. Bolded cells indicate nonoverlapping scheme

	Environment					
Genotypes	1	2	3	4	5	6
1	**x**	x	x	x	x	x
2						
3	**x**					
4	**x**					
5						
6						
7		**x**				
8		**x**				
9						
10						
11			**x**			
12			**x**			
13						
14	x	x	x	**x**	x	x
15				**x**		
16				**x**		
17						
18						
19					**x**	
20					**x**	
21						
22						
23						**x**
24						**x**
Total testing set	4	4	4	4	4	4
Total prediction set	20	20	20	20	20	20

### 2.4 Statistical models

The main objective of this research was to assess the genomic‐based prediction accuracy of several allocations of wheat lines in environments for NOL/OLO allocation of lines. Based on this objective and on the fact that independent random CV needs to be done for studying the different prediction abilities of the various NOL/OL allocation designs, a two‐stage unweighted analysis was used to adequately account for the within‐environmental variance in the first stage and the genomic effect and the genotype × environment effect in the second stage (Damesa et al., 2019). The first‐stage analyses consist of computing the best linear unbiased estimators (BLUEs) of all the lines for each of the environments. We used ASReml for R for a mixed model analysis (Butler et al., 2009) for grain yield in each environment‐year combination. The model used to calculate the BLUEs of the lines for each environment was:

$$\;y_{jkb}=\;\mu +L_{j}+r_{k}+ib{\left(r\right)}_{b}+e_{jkb}$$

where *y_jkb_* represents the phenotypic trait analyzed (grain yield) on the *k*th replicateof the *j*th line in the *b*th incomplete block, μ is an intercept, *L_j_* is the fixed effect of the *j*th wheat line, *r_k_* is the random effect of the *k*th replicate that is independently, identically, and normally distributed (iid), such that *r* = {*r_k_*} ∼ *N*(0, $I\sigma_{r}^{2}$) (where **I** is the identity matrix and $\sigma_{r}^{2}$ is the variance among replicates), *ib*(*r*)*_b_* denotes the random effect of the *b*th incomplete block within the *k*th replicate assumed to be iid and normally distributed such that $ib\left(r\right)\;=\;\left\{\;ib{\left(r\right)}_{b}\right\}∼$ *N*(0,$\;I\sigma_{ib(r)}^{2}$) with $\sigma_{ib(r)}^{2}$ being the variance of the incomplete block within each replicate, and *e_jkb_* is the random error assumed to be iid and also normally distributed such that **e** = {*e_jkb_*} ∼*N*(0,$\;I\sigma_{e}^{2}$), where$\;\sigma_{e}^{2}$ denotes the error variance. In this model, the line effect and its interaction with environments cannot be separated. Note that adding the design effects with randomized complete blocks, or any type of incomplete block design, does not pose a problem. In addition, modeling the residuals by spatial analyses to further control local variability does not present any additional difficulty. Thus, in the first‐stage of the analyses, the aim is to account for all environmental effects existing in each environment (site) such that the BLUEs of each wheat lines can be used for the combined analyses across environments for modeling GE including genomic information.

To implement the second‐stage GP analysis, we used the reaction norm model (Jarquín et al., 2014) that is an extension of the random effect genomic best linear unbiased predictor model, in which the main effect of lines, environments, markers, and their interactions are modeled using random‐effects (variance‐) covariance structures that are functions of genomic and environmental factors. Note that in the model above, the effect of the line (*L_j_*) can be replaced by *g_j_*, which is an approximation of the genetic value of the *j*th line derived from the genomic relationship matrix. In the models described below, we used *g_j_* along with its interaction with the environment *E_i_* (*gE_ij_*). These terms are further defined in the next section. The efficiency of the different allocation designs for sparse testing methods previously described was evaluated based on the genome‐enabled prediction accuracy of the three models described below.

#### 2.4.1 M1: E+L

This is similar to the first‐stage model described above, where *L_j_* and its interaction with environment cannot be separated. In this model, the response variable corresponds to the adjusted phenotypes (*y_ij_*) (BLUE) obtained after fitting the corresponding model for the incomplete block design described previously. In this case, the environment and line effects are considered as random. The linear model can be described as follows:

(1) $$y_{ij}=\;\mu +E_{i}+L_{j}+e_{ij}$$

where *E_i_* is the random effect of the *i*th environment, *L_j_* is the random effect of the *j*th line, such that $E_{i}\overset{iid}{∼}N\left(0,\sigma_{E}^{2}\right)$, $L_{j}\overset{iid}{∼}N\left(0,\sigma_{L}^{2}\right)$, and *e_ij_* is the average error, assumed to be iid following normal densities *N*($0,\;\sigma_{e}^{2}$), where $\sigma_{e}^{2}\;$is the within‐environment error variance, assumed to be constant.

#### 2.4.2 M2: E+L+G

This model is an extension of M1, and it considers the inclusion of the genomic random effect of the lines *g_j_*, which is an approximation of the genetic value of the jth line. This model component can be defined by the regression on *p* marker covariates,

$$g_{j}=\sum_{m=1}^{p}x_{jm}b_{m}\;$$

where *x_jm_* is the genotype of the *j*th line at the *m*th marker, and *b_m_* is the effect of the *m*th marker. Assuming that $b_{m}\overset{iid}{∼}N\left(0,\sigma_{b}^{2}\right)$ (*m = *1, …, p*)* and with $\sigma_{b}^{2}$ being the variance of the marker effects, the vector of genomic effects $g\;=\{g_{j}\}\;$ follows a multivariate normal density with zero mean and variance‐covariance matrix $\mathrm{Cov}\;\left(g\right)=\;G\sigma_{g}^{2}$ such that $g∼N(0,G\sigma_{g}^{2})$ where $G\propto \frac{{\mathrm{XX}}^{\prime }}{p}$ is the genomic relationship matrix, Xis the centered and standardized (by columns) matrix of molecular markers and $\sigma_{g}^{2}\propto \sigma_{b}^{2}$ is the genomic variance. The resulting model is

(2) $$y_{ij}=\;\mu +E_{i}+L_{j}+g_{j}+e_{ij}$$

where the line effects of the vector of genomic random effects **g** are correlated such that M2 allows the borrowing of information across lines. A disadvantage of this model is that the genomic estimated breeding value for a line tested in different environments is the same, regardless of the environment. To allow a specific genomic effect in each environment, the interaction term (GE) is included in the following model.

#### 2.4.3 M3: E+L+G+GE

This model is an extension of the previous model that includes the GE using covariance structures as shown by Jarquin et al. (2014), such that the vector containing the GE effects **gE** = {*gE_ij_*} was modeled as follows: 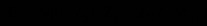 where the **Z_g_** and **Z_E_** are the incidence matrices to connect the phenotypes with genotypes and the environments, respectively; $\sigma_{gE}^{2}$ is the variance component of $gE_{ij}$, and “°” represents the Hadamard product (element‐by‐element product) between two matrices.

Combining the previous assumptions, the resulting linear predictor becomes

(3) $$y_{ij}=\;\mu +E_{i}+L_{j}+g_{j}+gE_{ij}+e_{ij}$$

where the *gE_ij_* term corresponds to the interaction between the genetic value of the *j*th genotype in the *i*th environment. Conceptually, this model term includes the interaction between each molecular marker and each environment. Because of the addition of environmental factors, this model can also be viewed as a reaction norm model that considers a main effect of the markers across environment and specific effects (slopes) for each environment.

### 2.5 Prediction assessment by random CV

In several genome‐enabled predictions studies two main random CVs are considered. Random CV1 includes certain proportion of lines never observed in any environments (Jarquin, et al., 2014). Random CV2 evaluates the prediction accuracy of models when some lines have been evaluated in some environments but not in others. In the CV2 prediction scheme, information from related lines (including the targeted line tested in other environments) and correlated environments (including the targeted environment) is used, and prediction assessment benefits from borrowing information between lines within an environment, between lines across environments, and among correlated environments (Burgueño et al., 2012).

The procedure to allocate the testing set in environments where no overlapped sets are considered is a particular case of the CV2 scheme with genotypes observed exclusively in only one environment. In this study, we considered a comprehensive and exhaustive set of OL genotypes across environments varying between 6 and 98%. A second CV scheme (CV1) considers the problem of predicting newly developed lines that have not yet been observed in any fields, and this scheme corresponds to the case of 100% OL genotypes across environments. Here, the prediction accuracy relies mostly on the genomic relationships between lines in the training and testing sets.

The prediction accuracy was measured on a trial basis as the Pearson correlation coefficient between the observed and predicted values within environments. For each period, the sample sizes of the genotypes in the prediction set within environments were different: 785 (W1; 942/6 × 5), 840 (W2; 1,008/6 × 5), and 865 (W3; 1,038/6 ×5).

All CV schemes were evaluated using 25 initial random partitions to initially split the full phenotypic data set into training and testing sets. Then, systematically different allocation compositions of the nonoverlapped/overlapped ratio were built, also considering the reduction of the training set size by sets of 10 genotypes. The variance components were computed for each random training partition set within each allocation composition and then averaged by allocation composition. In addition, the average percentage of the phenotypic variability explained by the genomic related terms from M2 (G) and M3 (G+G×E) were computed as the sum of all variance components of random effects, and the variance of a random effect is taken to be the variance explained by that effect.

We computed the genomic‐prediction accuracy of each model for each environment and each data set and presented the average of the genome‐enabled accuracies across environments for each of the three data sets.

### 2.6 Data availability and software

The six data files comprising three phenotypic data sets and three genomic data sets can be downloaded using the link https://hdl.handle.net/11529/10548552. The genomic prediction analyses were performed using R (R Development Core Team, 2020) and the models were fitted using the BGLR package (Pérez & de los Campos, 2014). Models were fitted considering 24,000 MCMC iterations with a thinning of 5. The first 4,000 iterations were used as burn‐in.

## 3 RESULTS

After applying conventional quality control (discarding SNPs with more than 50% of missing values and with minor allele frequency smaller than 3%) on the molecular markers, the number of SNPs remaining in the analysis were 6,071 (W1), 5,963 (W2), and 8,312 (W3).

Because of the extensive number of cases needed to combine different allocation sizes and compositions (ratio) of the nonoverlapped/overlapped allocation combinations considered in this study, only certain representative combinations of sizes and nonoverlapped/overlapped allocation compositions are presented.

### 3.1 Percentage of variance accounted for by the different components of the three genome‐enabled prediction models

**FIGURE 1:**
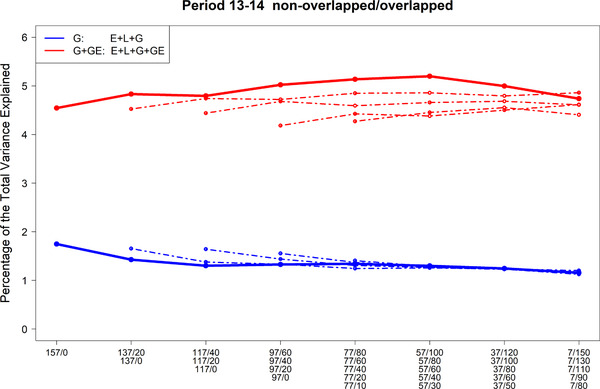
Wheat period 2013–2014. Percentage of the total variance explained by G (genomic) and GE (genomic × environment interaction) for the models M2 (E+L+G), and M3 (E+L+G+GE) for different sizes and compositions of the nonoverlapped/overlapped allocation designs. The solid blue line denotes the mean variance component of G from model M2 (E+L+G), whereas dashed‐dotted blue lines are the mean variance components of G from model M2 (E+L+G) for different sizes and compositions of the nonoverlapped/overlapped allocation designs. The solid red line denotes the mean variance component of G+GE from model M3 (E+L+G+GE), whereas dashed‐dotted red lines are the mean variance components of G+GE from model M3 (E+L+G+GE) for different sizes and compositions of the nonoverlapped/overlapped allocation designs. The *x*‐axis shows the different sizes (157, 137, …, 77) and composition of the nonoverlapped/overlapped (NOL/OL ratio) allocation design for each of the size (e.g., for size 157, the NOL/OL ratios are 157:0, 137:20, …, 7:150)

**FIGURE 2:**
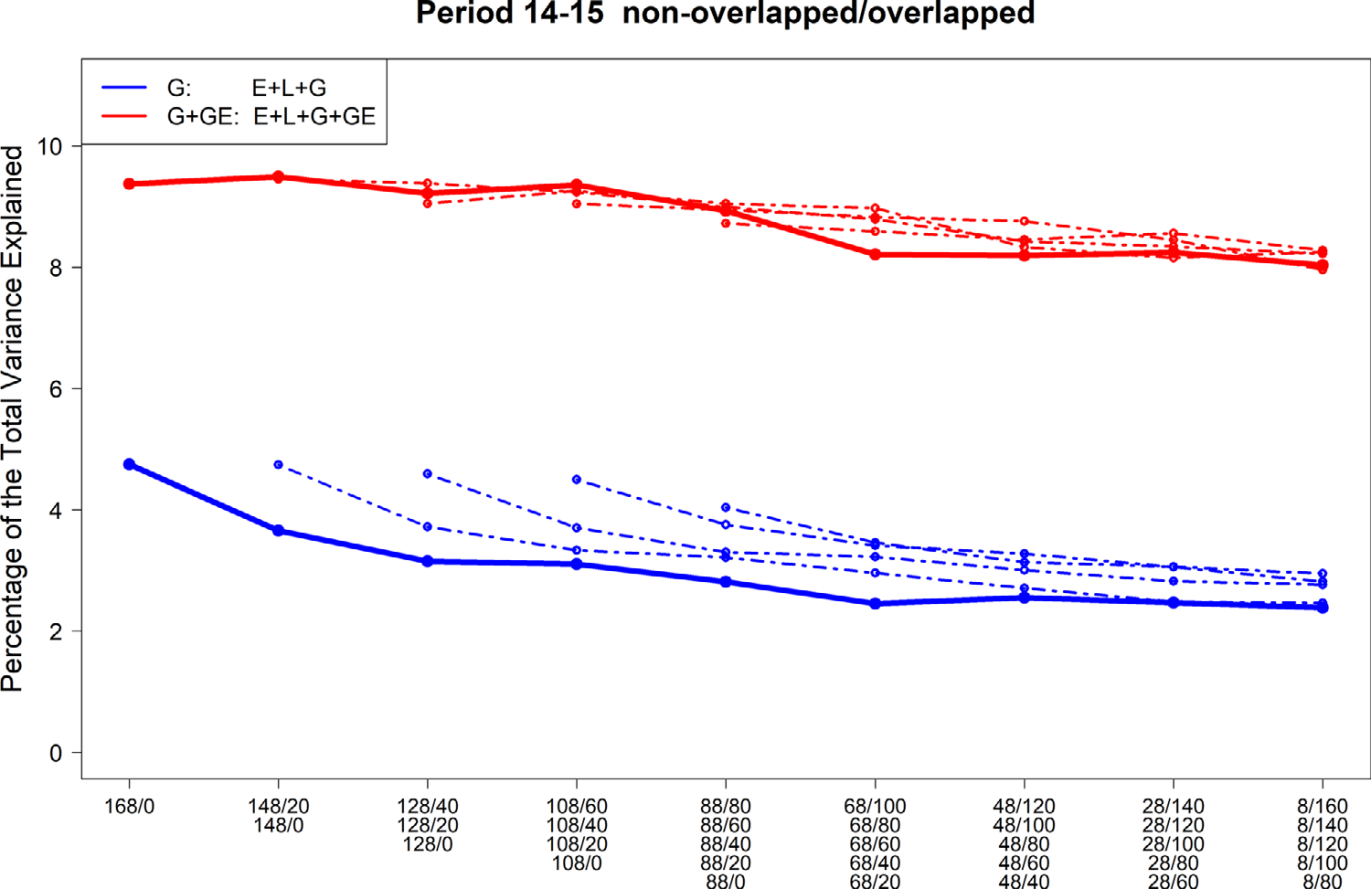
Wheat period 2014–2015. Percentage of the total variance explained by G (genomic) and GE (genomic × environment interaction) for the models M2 (E+L+G), and M3 (E+L+G+GE) for different sizes and compositions of the nonoverlapped/overlapped allocation designs. The solid blue line denotes the mean variance component of G from model M2 (E+L+G), whereas dashed‐dotted blue lines are the mean variance components of G from model M2 (E+L+G) for different sizes and compositions of the nonoverlapped/overlapped allocation designs. The solid red line denotes the mean variance component of G+GE from model M3 (E+L+G+GE), whereas dashed‐dotted red lines are the mean variance components of G+GE from model M3 (E+L+G+GE) for different sizes and compositions of the nonoverlapped/overlapped allocation designs. The *x*‐axis shows the different sizes (168, 148, …, 88) and composition of the nonoverlapped/overlapped (NOL/OL ratio) allocation design for each of the size (e.g., for size 168, the NOL/OL ratios are 168:0, 148:20, …, 8:160)

**FIGURE 3:**
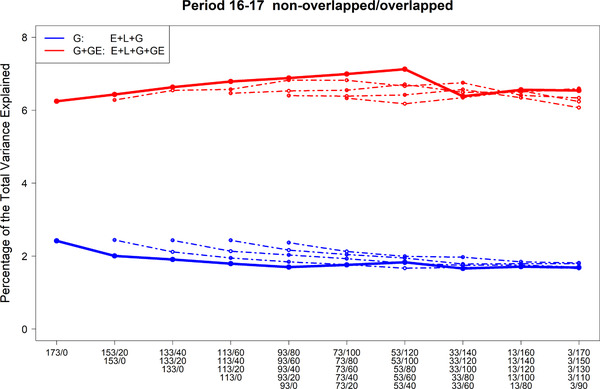
Wheat period 2016–2017. Percentage of the total variance explained by G (genomic) and GE (genomic × environment interaction) for the models M2 (E+L+G), and M3 (E+L+G+GE) for different sizes and compositions of the nonoverlapped/overlapped allocation designs. The solid blue line denotes the mean variance component of G from model M2 (E+L+G), whereas dashed‐dotted blue lines are the mean variance components of G from model M2 (E+L+G) for different sizes and compositions of the nonoverlapped/overlapped allocation designs. The solid red line denotes the mean variance component of G+GE from model M3 (E+L+G+GE), whereas dashed‐dotted red lines are the mean variance components of G+GE from model M3 (E+L+G+GE) for different sizes and compositions of the nonoverlapped/overlapped allocation designs. The *x*‐axis shows the different sizes (173, 153, …, 93) and composition of the nonoverlapped/overlapped (NOL/OL ratio) allocation design for each of the size (e.g., for size 173, the NOL/OL ratios are 173:0, 153:20, …, 3:170)

Figure 1 (W1), Figure 2 (W2), and Figure 3 (W3) display the average percentage of the variability explained by the genetic components of the described prediction models M2 and M3.

The variance component for the G term from model M2 (solid and dashed blue lines) explains the lowest percentage of variability for most of the ratio of NOL/OL allocations and ranged from around 2 to 4% (Figures 1, 2, 3). Only for period 14–15 (Figure 2), when the ratio of NOL/OL lines was 168:0, the percentage of variance explained by G in M2 was about 4.5% and it decreases slightly, approaching 2.5% as the number of unique wheat lines in the testing set was reduced (middle and right‐hand side of Figure 2). In general, for the three periods, the percentage of variance explained by G of M2 converged to the same values (around 2–2.5%) for all sizes of the testing sets when the ratio of NOL/OL lines is minimum.

The percentage of explained variability associated with the G+GE term in M3 is presented in the red solid and dashed‐dotted lines. However, the inclusion of GE increased the percentage of variability explained, in comparison with that in M2 (blue lines) for all three periods. The G+GE term of M3 explained over 4 to 9% of the total variability. Nonetheless, the G+GE of M3 displayed a slightly increased (or decreased) value on the proportion of variance explained as the number of common genotypes across environments was increased.

The size of the allocation design (Figures 1, 2, 3, solid lines vs. dashed‐dotted lines of the same color) impacted the ability of the model components to capture the signal from the total variance as the number of OL lines increased, particularly for GE model M3. It is interesting to observe that when the sample size decreases (see red dashed‐dotted lines), the percentage of variance of GE explained by model M3 stayed unchanged for the different NOL/OL ratios. The pattern for M2 is slightly different as NOL/OL increases.

In summary, for all three data sets, the G+GE components of model M3 captured the largest proportion of variance, and this pattern does not change much when the composition of the NOL/OL partitioning tends to decrease and thus a larger number of wheat lines are common in all of the environments; this result is also clear for smaller size samples. These results of the percentage of variance captured by G+GE are expected to be reflected in the genome‐enabled accuracy of the GE model (M3) and when more common lines are tested in all environments.

### 3.2 GP accuracy for the wheat data sets

**FIGURE 4:**
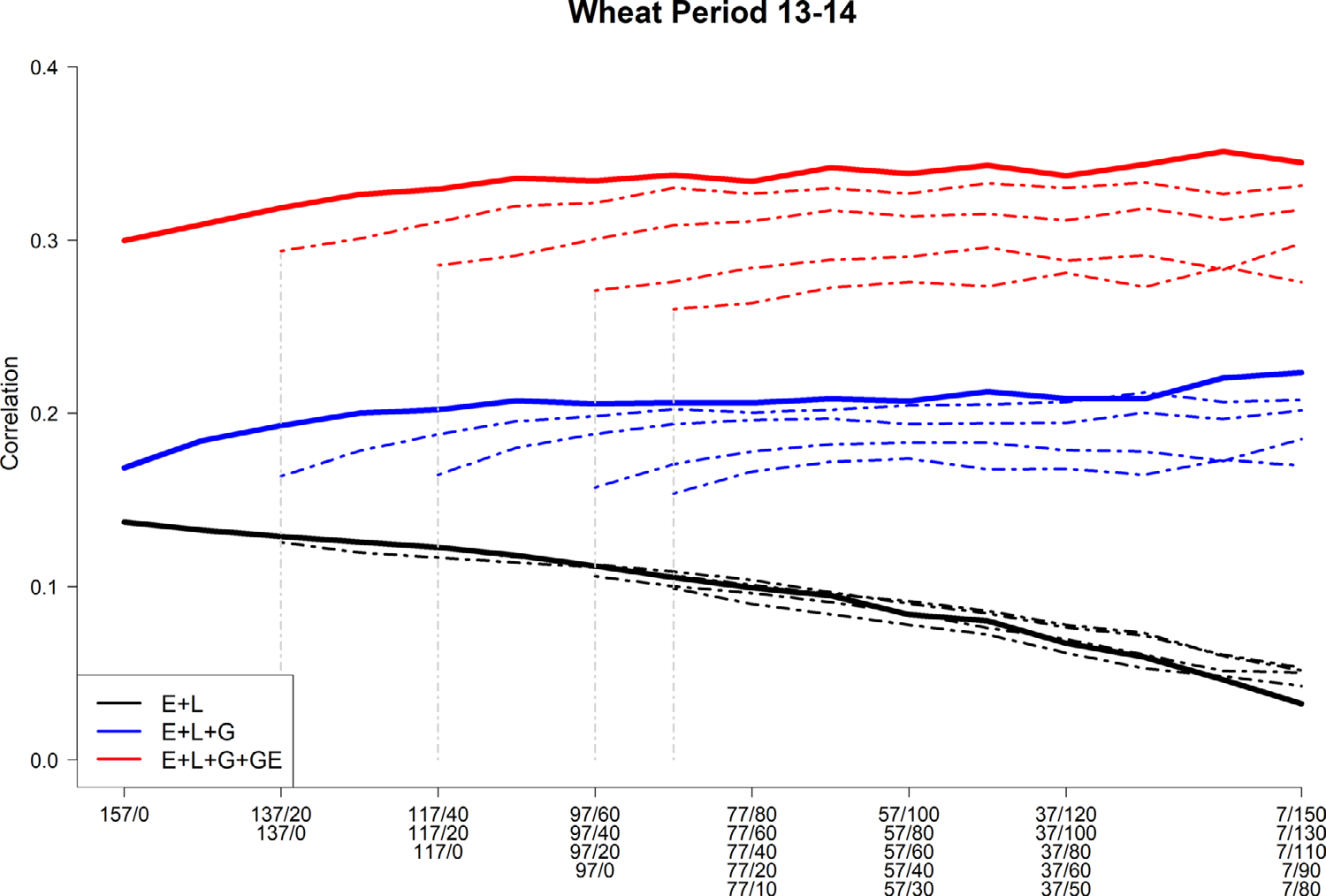
Wheat period 2013–2014. Average Pearson's correlation between the observed and predicted values of the wheat lines for models M1 (E+L) (black), M2 (E+L+G) (blue), and M3 (E+L+G+GE) (red) for different sizes and compositions of the nonoverlapped/overlapped allocation designs. Solid and dashed‐dotted red, blue, and black lines represent the mean for different sizes and compositions of the allocation designs for the same model (same color). The *x*‐axis shows the different sizes (157, 137, …, 77) and composition of the nonoverlapped/overlapped (NOL/OL ratio) allocation design for each of the size (e.g., for size 157, the NOL/OL ratios are 157:0, 137:20, …, 7:150)

**FIGURE 5:**
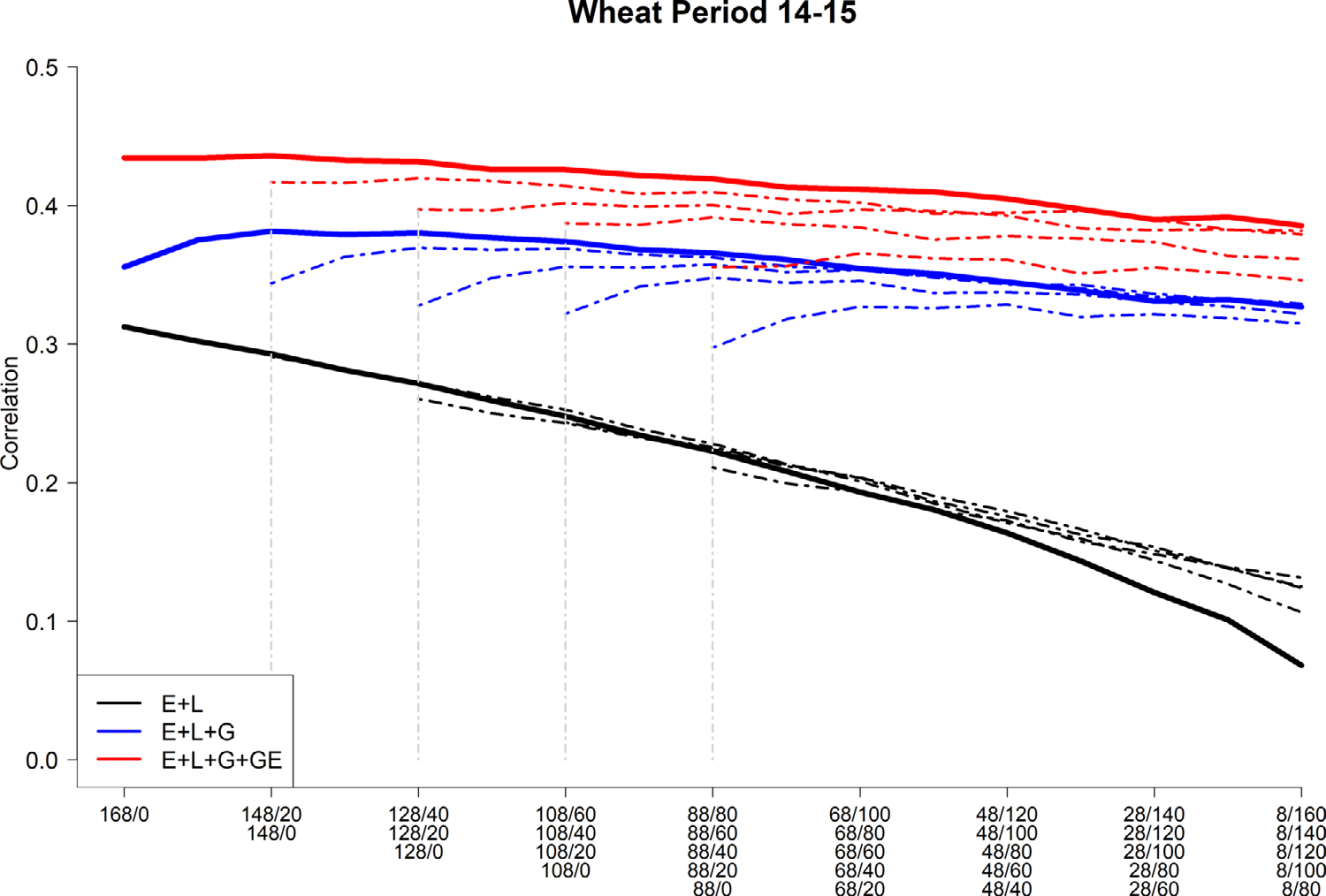
Wheat period 2014–2015. Average Pearson's correlation between the observed and predicted values of the wheat lines for models M1 (E+L) (black), M2 (E+L+G) (blue), and M3 (E+L+G+GE) (red) for different sizes and compositions of the nonoverlapped/overlapped allocation designs. Solid and dashed‐dotted red, blue, and black lines represent the mean for different sizes and compositions of the allocation designs for the same model (same color). The *x*‐axis shows the different sizes (168, 148, …, 88) and composition of the nonoverlapped/overlapped (NOL/OL ratio) allocation design for each of the size (e.g., for size 168, the NOL/OL ratios are 168:0, 148:20, …, 8:160)

**FIGURE 6:**
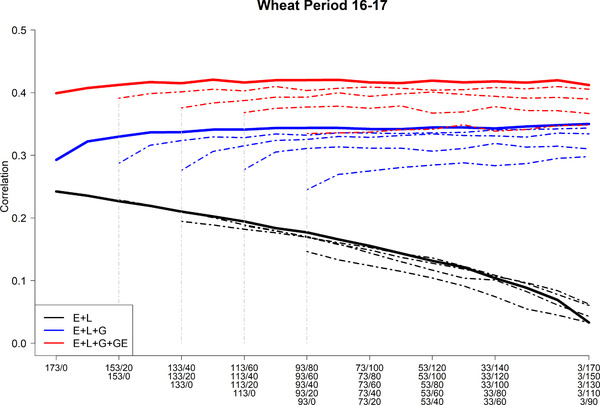
Wheat period 2016–2017. Average Pearson's correlation between the observed and predicted values of the wheat lines for models M1 (E+L) (black), M2 (E+L+G) (blue), and M3 (E+L+G+GE) (red) for different sizes and compositions of the nonoverlapped/overlapped allocation designs. Solid and dashed‐dotted red, blue and black lines represent the mean for different sizes and compositions of the allocation designs for the same model (same color). The x‐axis shows the different sizes (173, 153, …, 93) and composition of the nonoverlapped/overlapped (NOL/OL ration) allocation design for each of the size (e.g., for size 173, the NOL/OL ratios are 173:0, 153:20, …, 3:170)

Figures 4, 5, 6 show the average prediction accuracy across 25 random partitions for different size and composition configurations of calibration sets W1 (Figure 4), W2 (Figure 5), and W3 (Figure 6). In all periods, the same six environments were observed and the correlations for each combination (sample size × training‐testing partition × model × replicate) were computed on a trial basis (i.e., the correlation between predicted and observed genotypes was computed within environments). Then, the correlations were averaged across replicates within environments.

In general, similar trends in prediction accuracy were observed for all data sets and environments, with only slight variations. Reaction norm model M3 (red line) systematically returned the highest correlations, followed by M2 (blue line), which produced intermediate results, and M1 had the poorest predictive ability (Figures 4, 5, 6). These results indicate that the genome‐enabled models perform better when using the largest possible data set to calibrate models and when GE is modeled.

With regard to the effects of prediction accuracy by considering different ways to compose calibration sets, we observed that M3 produced at least as good results using OL sets of genotypes across environments than when the lines were observed only once (zero OL lines). Correlation values for M1 decreased as the number of OL genotypes was increased. This was expected because no genomic information was used to connect the genotypes in the calibration and prediction sets, so the prediction accuracy of unobserved genotypes in a particular environment relied on the phenotypic information of these genotypes observed in other environments. The prediction accuracies obtained for M1 were highest when all genotypes were observed only once in any of the environments (left side of the plots), whereas in the other side of the plot (right side), the correlations reached values near to zero. This is because on the right side of the plot the predictions are done randomly, since the model predicts genotypes that had never been observed using unrelated phenotypes due to the absence of covariance structures that link the observed and nonobserved genotypes.

For periods 2013–2014 and 2016–2017, and for some environments of period 2014–2015, the M2 model (blue) showed slight improvements with an increased number of common genotypes across environments. However, these results were within those obtained by M1 and M3 with the latter always outperforming the others for the same combinations between training set size and training composition (NOL/OL). Figure 5 (for period 2014–2015) show a slight decrease on the prediction accuracy for the different sample sizes and NOL/OL ratios as compared with the previous years.

Regarding the effects of decreasing the sample size for model calibration, a small reduction in predictive ability was observed for M2 and M3. However, in most of these cases, these values rose as the number of common genotypes across environments increased. In most of the cases for M1, the correlations decreased quickly with the reduction of the number of genotypes in the calibration sets and with the increase of the number of OL genotypes across environments.

The trends displayed in Figures 4, 5, 6 can be also observed for the values shown in Tables 2, 3, 4 for periods 2013–2014, 2014–2015, and 2016–2017, respectively. For period 2013–2014, for example, Table 2 shows that for NOL/OL lines of 157:0, the average correlation of model M3 was 0.300 and for the last option, 7:150, the average correlation was 0.345; the corresponding average correlations for model M2 were 0.169 and 0.223, respectively; meanwhile, for M1, the corresponding values were lower (0.137 and 0.032).

**TABLE 2:** Wheat period 2013–2014 (data set W1)

	157:0	137:20	117:40	97:60	87:70	67:90	47:110	27:130	7:150
**M1 = E+L**									
157	0.137	0.129	0.123	0.112	0.105	0.095	0.080	0.059	0.032
137		0.126	0.117	0.111	0.106	0.096	0.086	0.073	0.052
117			0.122	0.113	0.109	0.097	0.085	0.072	0.053
97				0.106	0.100	0.091	0.076	0.061	0.050
77					0.099	0.084	0.072	0.053	0.043
**M2 = E+L+G**									
157	0.169	0.193	0.202	0.206	0.206	0.208	0.213	0.208	0.223
137		0.164	0.188	0.198	0.202	0.202	0.205	0.212	0.208
117			0.165	0.188	0.194	0.197	0.194	0.200	0.202
97				0.157	0.171	0.182	0.183	0.178	0.185
77					0.154	0.172	0.168	0.165	0.170
**M3 = E+L+G+GE**									
157	0.300	0.319	0.330	0.334	0.338	0.342	0.343	0.344	0.345
137		0.294	0.311	0.322	0.330	0.330	0.333	0.333	0.332
117			0.285	0.301	0.309	0.317	0.315	0.318	0.318
97				0.271	0.276	0.289	0.296	0.291	0.298
77					0.260	0.273	0.273	0.273	0.276

*Note*: Average (across six environments) Pearson correlations (25 replicates) between the observed and predicted values for three models (M1–M3) for different sizes (157, 137, …, 77) and the composition of the nonoverlapped/overlapped (NOL/OL) ratio for each size (e.g., for size 157, the NOL/OL ratios are 157:0, 137:20, …, 7:150). E, environmental main effects; L, line main effects; G, genomic main effects; GE, genotype × environmental interaction.

**TABLE 3:** Wheat period 2014–2015 (data set W2)

	168:0	148:20	128:40	108:60	88:80	68:100	48:120	28:140	8:160
**M1 = E+L**									
168	0.313	0.293	0.271	0.248	0.223	0.193	0.164	0.121	0.068
148		0.291	0.272	0.253	0.228	0.201	0.171	0.144	0.106
128			0.261	0.243	0.224	0.204	0.179	0.151	0.124
108				0.244	0.226	0.204	0.176	0.154	0.125
88					0.211	0.193	0.173	0.148	0.131
**M2 = E+L+G**									
168	0.356	0.381	0.380	0.374	0.366	0.354	0.345	0.331	0.327
148		0.344	0.370	0.369	0.363	0.354	0.343	0.336	0.328
128			0.328	0.356	0.357	0.353	0.345	0.334	0.329
108				0.322	0.348	0.345	0.338	0.330	0.322
88					0.298	0.327	0.328	0.322	0.315
**M3 = E+L+G+GE**									
168	0.434	0.436	0.432	0.426	0.419	0.412	0.405	0.390	0.386
148		0.417	0.420	0.414	0.410	0.402	0.395	0.390	0.382
128			0.397	0.402	0.400	0.397	0.393	0.382	0.379
108				0.387	0.391	0.384	0.378	0.374	0.361
88					0.356	0.366	0.361	0.355	0.346

*Note*: Average (across six environments) Pearson correlations (25 replicates) between the observed and predictive values for three models (M1–M3) for different sizes (168, 148, …, 88) and the composition of the nonoverlapped/overlapped (NOL/OL) ratio for each size (e.g., for size 168, the NOL/OL ratios are 168:0, 148:20, …, 8:160). E, environmental main effects; L, line main effects; G, genomic main effects; GE, genotype × environmental interaction.

**TABLE 4:** Wheat period 2016–2017 (data set W3)

	173:0	153:20	133:40	113:60	93:80	73:100	53:120	33:140	3:170
**M1 = E+L**									
173	0.242	0.227	0.210	0.194	0.177	0.155	0.131	0.104	0.033
153		0.229	0.211	0.189	0.169	0.149	0.128	0.107	0.063
133			0.195	0.182	0.169	0.153	0.136	0.108	0.061
113				0.188	0.170	0.144	0.117	0.101	0.043
93					0.146	0.124	0.104	0.074	0.033
**M2 = E+L+G**									
173	0.293	0.330	0.337	0.341	0.344	0.342	0.344	0.343	0.350
153		0.287	0.324	0.328	0.332	0.336	0.336	0.340	0.343
133			0.276	0.315	0.325	0.329	0.334	0.331	0.334
113				0.277	0.311	0.311	0.307	0.319	0.310
93					0.245	0.275	0.284	0.283	0.298
**M3 = E+L+G+GE**									
173	0.399	0.412	0.415	0.416	0.420	0.416	0.419	0.418	0.412
153		0.391	0.401	0.403	0.403	0.409	0.404	0.408	0.405
133			0.376	0.387	0.393	0.394	0.401	0.394	0.390
113				0.368	0.377	0.375	0.367	0.378	0.367
93					0.335	0.337	0.341	0.339	0.348

*Note*: Average (across six environments) Pearson correlations (25 replicates) between the observed and predicted values for three models (M1–M3) for different sizes (173, 153, …, 93) and the composition of the nonoverlapped/overlapped (NOL/OL) ratio for each size (e.g., for size 173, the NOL/OL ratios are 173:0, 153:20, …, 3:170). E, environmental main effects; L, line main effects; G, genomic main effects; GE, genotype × environmental interaction.

For periods 2014–2015 (Table 3) and 2016–2017 (Table 4), the trends of the values of the genome‐enabled prediction accuracy were similar. The average Pearson correlations between observed and predicted values achieved accuracies ranging between 0.346–0.434 for model M3 in both periods. The superiority of M3 was observed across all sizes and composition of the NOL/OL allocation designs for the three periods. Small differences in genome‐enabled prediction accuracies could be found on the extremes of the ratio of NOL/OL lines. For example, for period 2014–2015, in the case of 168:0, the prediction accuracy was 0.434, whereas for the other extreme option of 8:160, the prediction accuracy decreased to 0.386 (Table 3). For period 2016–2017, the genome‐enabled prediction accuracy was 0.399 for 173:0 with a slight prediction increase when the NOL/OL ratio increased (0.412 for 153/20). On the other hand, for smaller NOL/OL ratios of 53:120, 33:140, and 3:170, the genome‐enabled prediction accuracies of M3 were 0.419, 0.418, and 0.412, respectively. (Table 4).

In summary, for the three wheat data sets, model M3 displayed the highest predictive ability, followed by model M2, while, as expected, M1 showed the poorest performance. These results were not significantly influenced by the size and the composition of the allocation designs. In most cases, the correlations between the observed and the predicted values were not highly affected by the magnitude of the ratio of NOL/OL lines of the lines in different environments, although slight decreases in prediction accuracy were observed for M3, especially in period 2014–2015. The genome‐enabled prediction accuracies of models M1 and M2 were consistently much lower than those obtained for model M3, including GE.

## 4 DISCUSSION

Studies focusing on the optimization of markers and field evaluation to increase genetic gains are crucial. Assuming a breeder has a certain number of lines and environments, there are different strategies in terms of how many lines to genotype and how many lines to phenotype in the given environments to maximize genetic gains for each strategy. Defining the fixed financial resources allows the researcher to define the cost of genotyping in terms of plot unit and to set different strategies in terms of the number of lines to genotype and the number of lines evaluated in the field using a sparse allocation scheme (Cullis et al., 2006; Cullis et al., 2020).

Given fixed resources, several strategies could be studied. One approach to a sparse testing scheme could focus on increasing the intensity of selections by increasing the number of testing lines. Another strategy could be to investigate how many candidates could be repeatedly tested in environments and how many could be planted in only one or a few environments using replicated and/or unreplicated field designs.

The optimization of breeding programs is paramount to maximize the rate of genetic gain. Such an optimization can be coupled with modern tools and technologies to modify breeding schemes and the testing strategies. Adequate testing strategies are fundamental to accurately recycle the parents that will give rise to a new breeding cycle. Sparse testing can be seen as the CV2 scheme (Crossa et al., 2017) in genomic prediction studies that, if applied in breeding programs, can lead to a higher selection intensity and larger environmental sampling, since the number of lines to be evaluated can be increased along with the number of environments in which the lines are tested (Haile et al., 2020). In this study we systematically assessed the effects for the genome‐enabled predictive ability for allocation designs in which a certain number of different wheat lines are distributed in different environments (NOL) and another set of lines is repeatedly observed in all environments (OL).

In plant breeding the definition of TPE is of fundamental importance for resource allocation (Kleinknecht et al., 2013) and the multi‐environment wheat trials analyzed at this stage of the CIMMYT wheat breeding program; selection decisions and further shipping of germplasm to the locations representing each TPE (Crespo et al., 2021) are needed for each of the environmental types included in this study. Certainly, in the current study, each environmental type (TPE) is represented by only a single trial or environment. However, for each type (e.g., Beds 5 irrigations), there is likely to be GE within the environments of the TPE. Thus, for another environment within the same TPE, ranking of genotypes may change, so that GE within each TPE is not captured by the current design at this stage of the wheat breeding trials.

Results of the three wheat MET data sets measured for genomic prediction accuracy indicate that important savings could be achieved by OL a small number of lines in all environments (∼20–30) and allocating the rest of the lines in a NOL design in different environments. This study showed that the decay of the prediction accuracy was less pronounced for the most complex model when the ratio of the NOL/OL lines decreased than for the simplest model. Clearly, the statistical models that included the GE component (M3) had an important effect on the final prediction accuracy by leveraging the interaction between genotypes and environments via other genetically related genotypes tested in these target environments. In this case, significant cost savings and increases in genome‐based accuracy can be achieved by testing more common lines in all the environments with model M3, which offers the flexibility of adapting to the seed availability in breeding programs, since for a fixed sample size, this model delivers similar levels of predictive ability. This model also offers the advantage of increasing the savings of testing in fields by delivering similar levels of predictive ability with reduced sample sizes.

In the three wheat data sets, the decrease in the size of the training set, represented by dashed‐dotted lines (in the figures), had, as expected, a negative effect on the prediction accuracy, yet when the ratio of NOL/OL lines decreased, better prediction accuracies were also achieved. These results can be explained by the large patterns of GE variance recovered by GE model M3. In general, genome‐enabled prediction accuracies follow similar trends, such as the variance captured by G and G+GE in the different models.

### 4.1 The genome‐enabled prediction models

In model M1, for the disjointed partition (NOL/OL) including zero OL wheat lines across environments, the effect of the environments can be confused with the line effect; therefore, the prediction of an unobserved line in a particular environment is mainly influenced by the single observation (replicate) of that line yet measured in a different environment. In this case, predictive ability strongly relies on phenotypic information of already‐tested genotypes.

The percentage of genomic variance explained by model M2 including genomic information is low in the three wheat data sets, with these values ranging close to 2–4% of the total captured variance. The genome‐enabled prediction accuracy is intermediate between model M1 and model M3. The main reason why model M3 was always the best predictive model is because the G+GE terms explained the larger percentage of the total variance. The GE included in model M3 allows the borrowing of information from related lines evaluated in correlated environments. This was partly possible because the E term involved in the GE component was modeled via the genetic correlation between environments. It should be pointed out that the variance–covariance structure of M3 was calculated separately for each of the random partitions in each replicate.

Other prediction models can be used to capture GE and predict unobserved lines. For example, the factor‐analytic model (Burgueño et al., 2012) used to capture the unstructured phenotypic variance–covariance matrix could be compared with the prediction accuracy results from model M3. In addition, in this study, we used only genomic information, although it might be possible to add pedigree information, incorporate it into M3, and thus potentially increase the prediction accuracy of the unobserved individuals in the designs with different allocations of NOL/OL lines. Further research for optimizing the NOL/OL can be achieved through computer simulation and applying a more flexible and parsimonious statistical models for assessing the complex phenomenon of GE under sparse testing.

### 4.2 Sparse field allocation of line methods for genomic selection

Plant breeding programs have limited financial resources per plot unit; therefore, planting only on a limited number of plots while optimizing the molecular and field evaluation resources with the objective of increasing genetic gains is of paramount importance. Hence, given fixed costs, breeders must study how many lines could be genotyped and how many of the total lines could be evaluated in the field, in order to design allocation methods that save on resources while increasing genetic gains. Some researchers aim to test more lines by using a sparse testing allocation method that focuses on increasing the intensity of selection, thus optimizing the response to selection. Other researchers prefer to maximize the genetic gains with a fixed plot unit cost but without increasing the intensity of selection, as enlarging field trials will inevitably increase the costs of phenotyping.

Results of this study show that important savings could be achieved by overlapping a small number of lines in all environments (∼30–50) and allocating the rest of the lines in in different environments. The statistical genome‐enabled prediction model that included the GE component (M3) had an important effect as it allows borrowing information from related lines tested in other environments. This offers cost savings, and it allows testing more common lines in all or some environments and by using a GE with model M3. Model M3 allows field testing savings while delivering similarly high levels of predictive ability under reduced sample sizes.

In addition, as pointed out, sparse testing schemes focusing on increasing the intensity of selection by increasing the number of tested lines will also increase the final genetic gains. In addition, our study is directly related to increases in genetic gains because we show how the genetic and GE variance components change with different NOL/OL. However, our study did not directly assess increasing the intensity of selection as a factor for increasing genetic gains. Our study did not directly study the effect of an unreplicated (augmented) design in terms of costs influencing the NOL/OL ratio. Despite this, some factors must be considered, and their study must leave research venues open. One aspect of unreplicated designs is that they facilitate the increase in population size and thus the intensity of selection, but at the cost of reducing the precision of the estimations. Another aspect of unreplicated designs is the necessary balance between plots assigned to unreplicated entries and plots with replicated entries (or checks). Genome‐enabled prediction accuracy usually requires good, solid, and extensive phenotype data of the lines in the testing set.

### 4.3 Single‐stage, stage‐wise, weighted, and unweighted analyses

In genome‐enabled prediction, data are usually obtained from multi‐environment trials and the analyses are conducted using a stage‐wise approach (Piepho et al., 2012; Schulz‐Streeck et al., 2013; Damesa et al., 2017, 2019). Application of stage‐wise analysis (and specifically a two‐stage analysis) is a valid procedure to account for the experimental design and thus control environmental variability (including plot‐to‐plot variability by means of spatial analyses) in the first stage and then performing the necessary random CVs for assessing the prediction accuracy of the different genomic models in the second stage. However, other stages can be considered before the one that accounts for random CV.

Further considerations could be necessary for controlling the heterogeneity of variances between trials and environments by performing a weighting method for the second stage where the weights are derived from the variances and covariances of adjusted means from the analysis of the previous stage. Damesa et al. (2019) mentioned there are different weighting methods: fully efficient variance‐covariance matrix (Piepho et al., 2012; Damesa et al., 2017) and diagonal weighting, with the diagonal elements of the inverse variance‐covariance matrix used as weights (Smith et al., 2001; Möhring & Piepho, 2009). Damesa et al. (2019) compared the weighted stage‐wise analysis versus unweighted stage‐wise analysis for GS using phenotypic and genotypic maize data; the authors found that the unweighted two or three‐stage method gave genome‐enabled prediction accuracy that was very similar to that of the weighted two or three‐stage method.

In this study the main objective was to examine the different NOL/OL allocations of wheat lines in environments and evaluate the genomic prediction ability of three models M1, M2, and M3. We performed genome‐enabled prediction using unweighted two‐stage analyses. We also ran weighted two‐stage analyses (data not shown) and obtained the same patterns of genomic prediction accuracy for the three models (M1, M2, and M3) under all NOL/OL allocation designs as those found in the weighted two‐stage analyses. The genome‐enabled prediction accuracies were, however, lower (∼10–25%) in the weighted two‐stage analyses than in the unweighted two‐stage analyses, although the NOL/OL patterns were the same in both methods. The year effect was noticed for period 2014–2015 where the predictions accuracy slightly decreased for all the sample sizes and the composition of the NOL/OL as compared with the previous years (periods) where the NOL/OL ratio showed a stable genome‐enabled prediction accuracy.

### 4.4 Bayesian priors and variance components

We fitted models M1–M3 using all available records to assess the proportion of the variance explained by environment, genotype, and GE interaction. Similar analyses have been done before, for example, Jarquín et al. (2014), Pérez‐Rodríguez et al. (2015) and, more recently, de los Campos et al. (2020), where the authors concluded that a big proportion of the grain yield variance is explained by environmental factors, followed by the GE interaction. Results obtained here are in agreement with those results. We have found that, regardless of the model, ∼82–93% of grain yield variance is explained by environmental factors (main effect of the environment), the residual variance explains between 5–10% of the phenotypic variance, and the rest of the terms in our models (G and GE) explains between ∼1–6% of the phenotypic variance. The GE has a sizable effect and explains between ∼3.3–5.7 of the phenotypic variance. These variances (proportions) are along the same lines with those accounting for G+GE at different values of the NOL/OL ratio shown in Figures 1, 2, 3.

The three models were fitted in the BGLR statistical package which uses MCMC techniques to fit the models, and prior distributions to the parameters of interest need to be specified. In the case of variance parameters, the software assigns independent inverse chi‐squared distributions to each variance parameter, and the hyper‐parameters are assigned so that the resulting prior distributions are proper but slightly informative. Hyperparameters are selected automatically using a prior partition of the phenotypic variance, and the rules are described clearly in Appendix B of the BGLR documentation (Pérez & de los Campos, 2014). The inverse scaled chi‐squared distribution is just an option for the prior distribution. Gelman (2006) has argued against these types of priors because they are sensitive to the selection of hyperparameters, which is true for small sample sizes, but it is well known that as the sample size increases, the likelihood dominates the prior. In our case, the sample size is big enough to estimate the variance parameters. A sensitivity analysis of the prior in the fitted models is out of the scope of this study, where the main interest is the prediction ability of the models.

### 4.5 Design approach to improve sparse testing allocation under genomic‐based relationships

Modern plant breeding requires the use of linear mixed models with treatment (genotypic) effects as random, where the association between cultivars is measured either by pedigree or by genomic information. Linear mixed model procedures for developing incomplete block or other complex blocking structure designs can be used that will take into account the pedigree of genomic relationships (Piepho et al., 2020). Genome‐enabled prediction in genomic‐assisted breeding programs opens up questions regarding how to select individuals for phenotyping in several environments based on genomic data and thus increase the efficiency of plant breeding. Heslot and Feoktistov (2020) presented optimization methods that offer gains in precision when evaluating selection candidates under genomics.

The sparse testing allocation presented in this study as well as in Jarquín et al. (2020) could be further optimized by regarding each environment as an incomplete block of a given size (the number of lines we will test in each environment) and thus finding an optimal incomplete block design. This could be improved further by including the pedigree or the genomic information of the tested lines. Research is required to find out if designs can be optimized for the G main effect, and thus improve the sparse designs currently considered.

## 5 CONCLUSIONS

This investigation examined and evaluated the genome‐enabled prediction accuracy in different field sparse testing systems consisting of different ratios of NOL/OL wheat lines included in environments. The genome‐based model, including GE, captured more genetic variability than the main genomic effects models. In addition, the genomic models with GE provided higher prediction accuracy than those models that did not include this interaction term in the different allocation designs comprising different combinations of NOL/OL lines in environments. Reducing the sample size of the lines assigned to environments decreased the genome‐enabled prediction accuracy, but these trends decrease when more lines are OL in all the environments; that is, the levels of genome‐enabled accuracy are recovered when the number of OL lines tested across environments increases. Models including GE offer the possibility of maintaining the prediction accuracy when both extreme situations take place—all NOL lines and all OL lines while reducing the size of the training set. Results show that important savings in testing resources could be achieved by optimizing the allocation of resources (lines tested in environments) using genome‐based models including GE. In general, for the three data sets used in this study we found that having a small proportion of lines (30–50) OL in all the environments with a sizeable proportion of lines NOL in the environments resulted in stable genome‐enabled prediction accuracies of unobserved lines in environments.

## AUTHOR CONTRIBUTIONS

Leonardo Crespo‐Herrera: Conceptualization; Investigation. Reka Howard: Conceptualization; Formal analysis; Investigation; Methodology. Hans‐Peter Piepho: Conceptualization; Investigation. Paulino Pérez‐Rodríguez: Conceptualization; Investigation. Osval Montesinos‐Lopez: Conceptualization; Investigation. Juan Burgueño: Conceptualization; Investigation; Methodology. Ravi Singh: Conceptualization; Data curation; Investigation; Methodology. Suchismita Mondal: Conceptualization; Data curation; Investigation; Methodology. Diego Jarquín: Conceptualization; Data curation; Formal analysis; Investigation; Methodology. Jose Crossa: Conceptualization; Investigation; Methodology.

## CONFLICT OF INTEREST

The authors declare no conflict of interest.
